# HIV Infection and Geographically Bound Transmission of Drug-Resistant Tuberculosis, Argentina

**DOI:** 10.3201/eid1811.120126

**Published:** 2012-11

**Authors:** Viviana Ritacco, Beatriz López, Marta Ambroggi, Domingo Palmero, Bernardo Salvadores, Elida Gravina, Eduardo Mazzeo, National TB Laboratory Network, Susana Imaz, Lucía Barrera

**Affiliations:** Instituto Nacional de Enfermedades Infecciosas ANLIS “Dr. Carlos G. Malbrán,” Buenos Aires, Argentina (V. Ritacco, B. López, E. Mazzeo, L. Barrera);; Hospital “Dr. F.J. Muñiz,” Buenos Aires (M. Ambroggi, D. Palmero);; Programa Provincial de Tuberculosis, Santa Fe, Argentina (B. Salvadores);; Hospital Zonal General Agudos “Dr. Diego Paroissien,” La Matanza, Argentina (E. Gravina);; and Instituto Nacional de Enfermedades Respiratorias “Dr. Emilio Coni” ANLIS “Dr Carlos G Malbran,” Santa Fe (S. Imaz)

**Keywords:** multidrug-resistant tuberculosis, extensively drug resistant tuberculosis, antimicrobial resistance, tuberculosis and other mycobacteria, TB, MDR TB, XDR TB, co-infection, HIV, genotype, Argentina, restriction fragment length polymorphism, geography, disease transmission

## Abstract

During 2003–2009, the National Tuberculosis (TB) Laboratory Network in Argentina gave 830 patients a new diagnosis of multidrug-resistant (MDR) TB and 53 a diagnosis of extensively drug- resistant (XDR) TB. HIV co-infection was involved in nearly one third of these cases. Strain genotyping showed that 7 major clusters gathered 56% of patients within restricted geographic areas. The 3 largest clusters corresponded to epidemic MDR TB strains that have been undergoing transmission for >10 years. The indigenous M strain accounted for 29% and 40% of MDR and XDR TB cases, respectively. Drug-resistant TB trends in Argentina are driven by spread of a few strains in hotspots where the rate of HIV infection is high. To curb transmission, the national TB program is focusing stringent interventions in these areas by strengthening infection control in large hospitals and prisons, expediting drug resistance detection, and streamlining information-sharing systems between HIV and TB programs.

## Abstract 2

Disease trends are driven by HIV co-infection and transmission of a few strains within narrow geographic niches.

During the early 1990s, HIV-associated multidrug-resistant tuberculosis (MDR TB) emerged in Argentina (*1*). In Buenos Aires, the country’s most heavily populated city, certain multidrug-resistant *Mycobacterium tuberculosis* strains spread quickly among patients with AIDS (*2*,*3*). Specifically, the so-called M strain caused a major MDR TB outbreak at the Hospital Muñiz, a referral treatment center for infectious diseases (*4*). HIV-infected patients repeatedly seeking assistance at different health centers introduced the M strain into hospitals in nearby districts, where secondary transmission occurred (*5*). This strain was later responsible for the emergence of MDR TB in HIV-negative patients who had not previously undergone TB treatment (*6*). In 2002, the M strain was isolated from 2 patients with extensively drug-resistant TB (XDR TB). Two other MDR TB outbreak strains, Ra and Rb, emerged in Rosario, the third largest city in Argentina, simultaneously with the M strain (*7*).

MDR TB emergence highlighted the need for a MDR/XDR TB surveillance system focused on incidence and transmission. In 2003, the National TB Laboratory Network launched a systematic registry of all incident MDR/XDR TB cases diagnosed throughout the country. The registry includes a genotype database for all MDR/XDR TB patients going back to the initial outbreaks and population studies. We present the findings of a 7-year follow-up study of MDR and XDR TB in Argentina, with emphasis on potential transmission events involving strains responsible for previous outbreaks.

## Materials and Methods

### Study Group

Isolates from all patients with newly diagnosed MDR or XDR TB from January 2003 through December 2009 were included in the study (1 isolate per patient, collected at time of diagnosis). MDR TB was defined as disease caused by *M. tuberculosis* resistant to at least isoniazid and rifampin and XDR TB as disease caused by MDR *M. tuberculosis* showing further resistance to any fluoroquinolone and any second-line injectable anti-TB drug. A patient with newly diagnosed MDR or XDR TB was defined as a patient with disease first confirmed by drug susceptibility testing (DST) during the study period, regardless of previous treatment history. A hotspot was defined as an area where a MDR TB outbreak had been documented before the study period. Two or more patients were considered to be epidemiologically related when they were in the same place and time or shared similar behavioral risk factors.

Available demographic and clinical data were collected through the national TB laboratory network. A special effort was made to retrieve data from clinical records in special groups, i.e., XDR TB, patients in hotspot areas, and those in clusters with <6 bands in the IS6110 restriction fragment length polymorphism (RFLP). This research was approved by the research review board of the Instituto Nacional de Enfermedades Infecciosas, Administración Nacional de Laboratorios e Institutos de Salud (INEI ANLIS) “Carlos G. Malbran.”

### Bacteriologic Studies

On the basis of programmatic guidelines, DST was performed on isolates from patients at risk for drug resistance: patients with TB treatment failure and retreatment, HIV or other concomitant conditions, or exposure to drug-resistant TB in household, prison, or hospital. In Buenos Aires and Rosario, culture and DST are available to test virtually all persons with suspected TB who seek assistance at large referral treatment centers. In the rest of the country, persons not included in the high-risk group are highly unlikely to contract MDR TB. In all, ≈10,000 TB cases are reported annually in Argentina, of which ≈4,500 are diagnosed on the basis of a positive culture. Among cases that are culture-positive, ≈3,000 have isolates submitted for DST; MDR TB is diagnosed for 4% of these patients.

*M. tuberculosis* DST to first-line drugs (isoniazid, rifampin, streptomycin, ethambutol, and pyrazinamide) was performed in 19 TB network laboratories under regular proficiency testing, according to World Health Organization standards (*8*). The supranational reference laboratory at Instituto Nacional de Enfermedades Infecciosas ANLIS conducted external quality control, confirmed multidrug resistance, and tested susceptibility to second-line drugs (kanamycin, amikacin, capreomycin, and ofloxacin), according to World Health Organization recommendations (*9*).

### Genotyping

All available isolates underwent standard IS*6110* DNA RFLP fingerprinting and spoligotyping (*10*,*11*). Patterns were compared by using BioNumerics 5.1 software (Applied Maths, St-Martens-Latem, Belgium), using the Dice coefficient with 1% tolerance and the unweighted pair-group method with arithmetic averages (*12*). The RFLP pattern of the reference strain *M. tuberculosis* Mt14323 was used for gel normalization.

For RFLP patterns with >6 bands, a cluster was defined as a group of >2 isolates whose RFLP patterns and spoligotypes were 100% identical when compared with all other patterns found within the study period. RFLP patterns with <6 bands were included in a cluster when epidemiologic links were established in addition to identical spoligotypes. Similarly, a proven epidemiologic link was required for including in a cluster a variant of a cluster genotype; a genotype variant was defined as a genotype with a 1-band difference in the RFLP or 1-spacer difference in the spoligotype but not both. Because of the unusually large size of some clusters, a major cluster was defined as >15 patients and a minor cluster as <15 patients with MDR TB newly diagnosed during the period. Shared International Type (SIT) and genotype family were assigned by consulting the SITVIT database (www.pasteur-guadeloupe.fr:8081/SITVIT) (*13*), except for genotypes H4, T1-Tuscany, and LAM3/S convergent, which were reclassified as Ural, LAM-Tuscany, and S, respectively, according to Abadia et al. (*14*). Orphan genotypes were those lacking a SIT in the SITVIT database.

### Statistical Analysis

We used univariate and multivariate logistic regression analyses to determine factors associated with being in a cluster and being in a major cluster. The explanatory variables were patient age, country of birth, place of diagnosis, HIV status, previous TB treatment, and disease localization. Within the subgroup included in major clusters, we used logistic regression analysis to determine factors associated with being infected by the M strain. In this latter model, the explanatory variables were patient age, country of birth, HIV status, previous TB treatment, hospital exposure, and isolate drug resistance.

We divided patients into 3 age groups: <15, 16–45, and >45 years of age. Gender was removed from the models because it was associated with particular settings in 2 major clusters; unknown categories were removed from all the variables included in the model. Because of the limited numbers per category, the age category <15 years was removed from the multivariate analyses.

We applied 3 tests to assess the performance of the models: overall model fit, Hosmer & Lemeshow test, and receiver operating characteristic area under the curve. We considered a model to be adequate when values were: overall model fit p<0.2, Hosmer & Lemeshow test p>0.5, and area under the curve >0.70. We used the χ^2^ test for linear trends for assessing changes in the annual number of MDR TB patients in cluster M compared with changes in numbers in other major clusters. Statistical analyses were performed by using MedCalc version 12 software (MedCalc, Mariakerke, Belgium).

## Results

### MDR TB Patients, Genotypes, and Clustering

#### Genotyping Coverage

**Figure 1:**
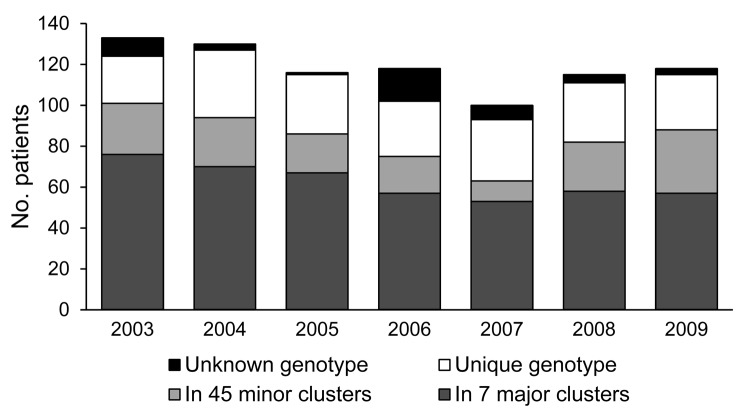
Numbers of patients with newly diagnosed multidrug-resistant tuberculosis reported per year, grouped according to genotype analysis, Argentina, 2003–2009. Major cluster, >15 patients; minor cluster, <15 patients.

Genotyping was available for isolates from 787/830 (94.8%) newly diagnosed MDR TB patients registered during the study period (2003, 93.2%; 2004, 97.7%; 2005, 99.1%; 2006, 86.4%; 2007, 93.0%; 2008, 96.5%; 2009, 97.5%) (Figure 1). Coverage was lower in 2006 because of a technical mishap that resulted in a loss in the isolate collection.

#### Genotype Family Distribution

The 3 predominant genotype families were LAM (38.8%), Haarlem (36.3%), and T (13.9%). Other genotypes were S (2.8%), U (1.7%), Beijing (1.5%), X (0.9%), and Ural (0.4%); orphan genotypes accounted for 3.8%. Within the 3 predominant genotype families, the most frequent subfamilies were H2 (29.5%), LAM3 (16.4%), T1 (8.9%), LAM5 (6.6%), LAM9 (6.5%), and Tuscany (5.5%). Of 12 patients carrying Beijing genotypes, 1 was born in Indonesia, 7 in Peru, and 4 in South America with no information on country of birth.

#### Clusters

Of 787 patients for whom isolate genotype was available, 438 (55.7%) fitted into 7 major clusters and 151 (19.2%) into 45 minor clusters; 198 (25.2%) harbored unique genotypes (Table 1). In the multivariate regression analysis, the outcome of being in a cluster was significantly dependent on being 15–44 years of age, born in Argentina, and HIV-infected. Being in a major cluster was significantly dependent on the 2 latter predictors and of having the MDR TB diagnosis occur in a hotspot area (Table 2).

**Table 1:** Patients with newly diagnosed multidrug-resistant TB, by year and genotype cluster, Argentina, 2003–2009

Genotype	No. patients							Total no. (%) patients
	2003	2004	2005	2006	2007	2008	2009	
Cluster M	46	40	33	29	31	28	21	228 (29.0)
Cluster Ra	13	6	19	12	15	14	10	89 (11.3)
Cluster Rb	7	10	2	1	5	6	7	38 (4.8)
Cluster Pr	4	5	4	4	1	3	5	26 (3.3)
Cluster At	3	4	1	1	1	4	7	21 (2.7)
Cluster Ob	2	1	5	5	0	1	4	18 (2.3)
Cluster Os	1	4	3	5	0	2	3	18 (2.3)
Minor cluster*	25	24	19	18	10	24	31	151 (19.2)
Unique	23	33	29	27	30	29	27	198 (25.2)
Total	124	127	115	102	93	111	115	787 (100.0)

*A total of 45 minor clusters were identified during the study period, each consisting of <15 new patients with multidrug-resistant tuberculosis.

**Table 2:** Predictors for being in cluster and in major cluster for 787 patients with multidrug-resistant TB, Argentina, 2003–2009*

Characteristic	No. patients	% Patients in cluster	Unadjusted OR (95% CI)	Adjusted OR (95% CI)†	% Patients in major cluster‡	Unadjusted OR (95% CI)	Adjusted OR (95% CI)§
Age, y, n = 640							
<15	24	83.3	2.9 (0.9–8.9)	ND	58.3	1.5 (0.6–3.6)	ND
16–45	495	78.6	**2.1 (1.4–3.2)**	**2.5 (1.3–5.0)**	57.9	1.5 (1.0–2.2)	1.0 (0.5–2.0)
>45	121	63.6	1	1	48.3	1	1
Country of birth, n = 541							
Argentina	412	80.1	**2.7 (1.7–3.9)**	**3.5 (1.9–6.4)**	66.7	**7.6 (4.7–12.1)**	**8.0 (4.3–15.0)**
Other	129	61.2	1	1	20.9	1	1
Place of diagnosis, n = 787							
Hotspot¶	634	77.9	**2.1 (1.5–3.1)**	1.6 (0.7–3.7)	62.3	**4.2 (2.9–6.2)**	**5.9 (2.5–13.8)**
Other	153	62.1	1	1	28.1	1	1
HIV status, n = 604							
Positive	254	86.6	**2.7 (1.7–4.1)**	**2.4 (1.0–5.6)**	76.4	**3.3 (2.-4.7)**	**3.7 (1.8–7.7)**
Negative	350	70.9	1	1	49.4	1	1
Previous TB, n = 557							
Yes	313	71.9	0.7 (0.5–1.0)	0.8 (0.5–1.5)	51.8	0.7 (0.5–1.0)	0.7 (0.4–1.2)
No	244	79.1	1	1	59.4	1	1
Site of disease, n = 775							
Pulmonary only	698	74.1	0.6 (0.3–1.2)	1.4 (0.5–4.2)	55.0	0.8 (0.5–1.3)	2.7 (1.1–7.0)
Other	77	81.8	1	1	59.7	1	1

*Boldface indicates significance. TB, tuberculosis; OR, odds ratio; ND, not done. †Cluster model overall model fit p < 0.0001, Hosmer & Lemeshow test p = 0.5888, area under the receiver operating characteristic curve 0.723. ‡Cluster including >15 patients in the study period. §Major cluster model overall model fit p < 0.0001, Hosmer & Lemeshow test p = 0.7766, area under the receiver operating characteristic curve 0.793. ¶Area where a multidrug-resistant outbreak was previously documented.

**Figure 2:**
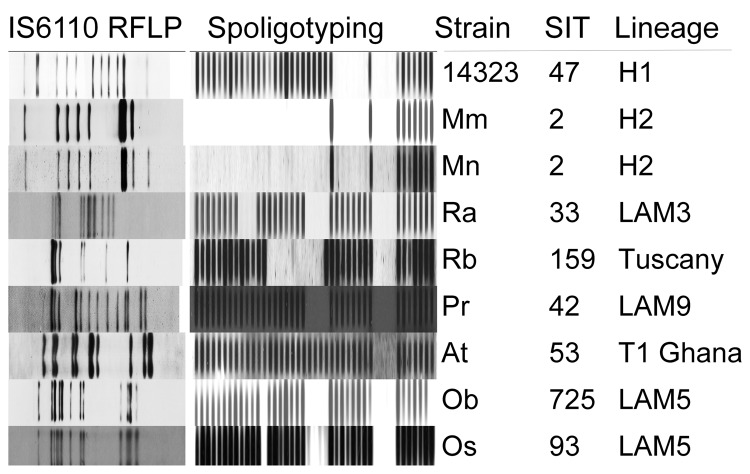
IS*6110* restriction fragment length polymorphism (RFLP) patterns and spoligotypes of 7 major cluster strains, including 2 main variants of M strain, and reference strain Mt 14323. SIT, Shared International Type in SITVIT database (www.pasteur-guadeloupe.fr:8081/SITVIT).

**Figure 3:**
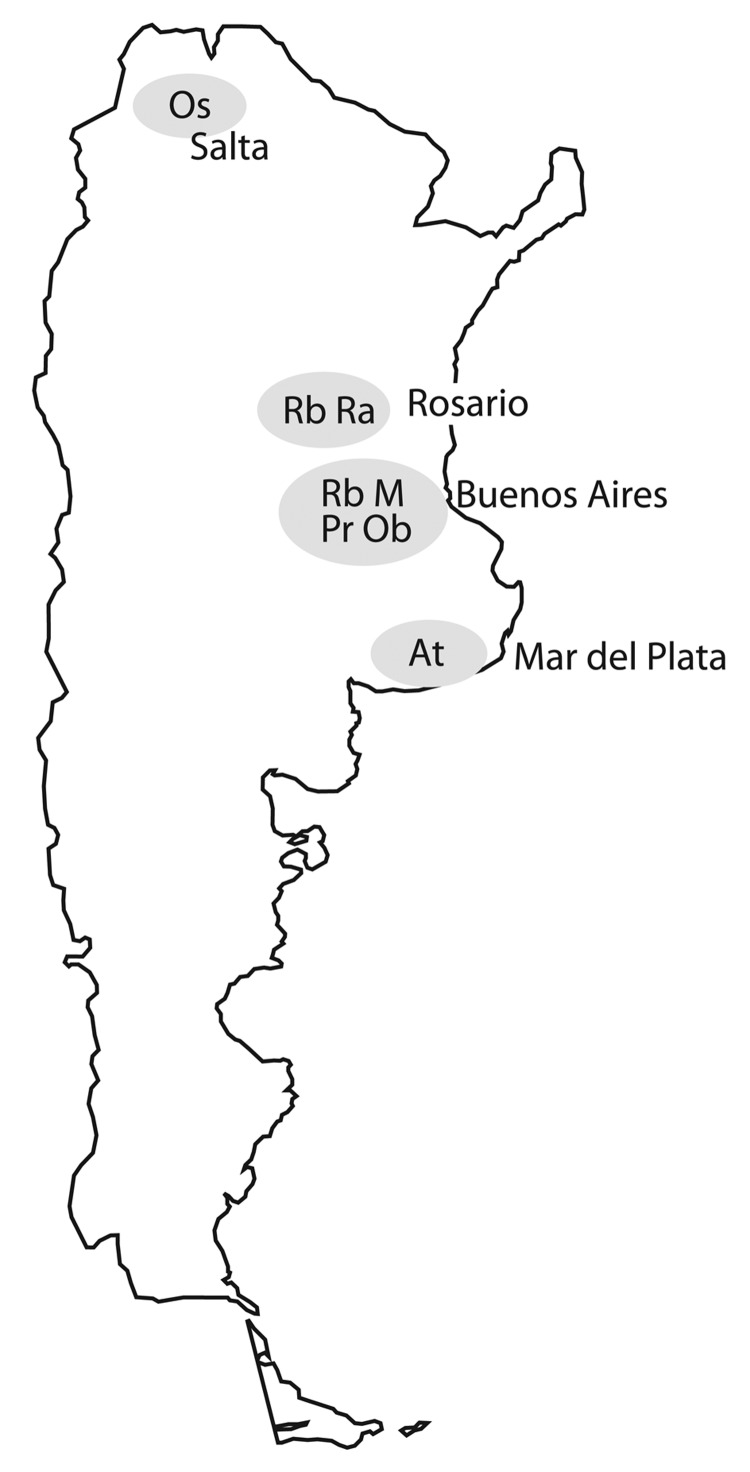
Locations of 7 major multidrug-resistant tuberculosis clusters, labeled by strain type, Argentina, 2003–2009.

Characteristics of the 7 major clusters are described in Table 3 and DST profiles in Table 4. Genotype patterns are shown in Figure 2 and geographic distribution in Figure 3. Altogether, the 3 largest clusters (M, Ra, and Rb) accounted for 355 (45.1%) patients; isolate patterns matched 3 genotypes previously associated with MDR TB outbreaks (*4*,*7*,*15*). The other 4 major clusters (Pr, At, Ob, and Os) accounted for 83 (10.5%) patients; these genotypes had been reported only sporadically before the study period.

**Table 3:** Putative risk factors for case-patients with newly diagnosed multidrug-resistant TB in 7 major genotype clusters, Argentina 2003–2009

Cluster (SIT)*	Area	Total no. case-patients	Risk factor, no. (%) case-patients						
			Previously treated for TB	HIV positive	HCWs	Other hospital exposure†	Prison	Household exposure	Unknown
M H2 (2)	Buenos Aries	228	78 (34.2)	116 (50.9)	21 (9.2)	38 (16.7)	23 (10.1)	29 (12.7)	19 (8.3)
Ra LAM3 (33)	Rosario	89	40 (44.9)	28 (31.5)	2 (2.2)	10 (11.2)	17 (19.1)	16 (18.0)	14 (15.7)
Rb Tuscany (159)	Buenos Aries, Rosario	38	11 (28.9)	22 (57.9)	0 (0.0)	1 (2.6)	0 (0.0)	13‡ (34.2)	6 (15.8)
Pr LAM9 (42)	Buenos Aries	26	10 (38.5)	10 (38.5)	0 (0.0)	2 (7.7)	15 (57.7)	5 (19.2)	2 (7.7)
At T1 (53)	Atlantic Coast	21	9 (42.9)	7 (33.3)	1 (4.8)	3 (14.3)	2 (9.5)	7 (33.3)	2 (9.5)
Ob LAM5 (725)	Buenos Aries	18	7 (38.9)	4 (22.2)	0 (0.0)	2 (11.1)	3 (16.7)	5 (27.8)	2 (11.1)
Os LAM5 (93)	Salta	18	7 (38.9)	6 (33.3)	0 (0.0)	3 (16.7)	1 (5.6)	9 (50.0)	2 (11.1)

*Sum does not equal total because of patients with more than one risk factor. Buenos Aires includes the city and surroundings. MDR, multidrug-resistant; TB, tuberculosis; SIT, Shared International Spoligo Type according to Brudey et al. (*13*); HCW, health care workers. †Previous hospitalization(s) or concomitant condition. ‡Ten of these case-patients shared a single residence with transvestite sex workers.

**Table 4:** Resistance of *Mycobacterium tuberculosis* isolates in 7 major clusters to antimicrobial drugs in addition to isoniazid and rifampin, Argentina, 2003–2009*

Cluster	Total no. isolates	No. (%) isolates with additional resistance to			
		0 drugs	1 drug	2 drugs	>3 drugs
M	228	2 (0.9)	13 (5.7)	30 (13.2)	183 (80.3)
Ra	89	8 (9.0)	61 (68.5)	15 (16.9)	5 (5.6)
Rb	38	26 (68.4)	6 (15.8)	4 (10.5)	2 (5.3)
Pr	26	26 (100)	0	0	0
At	21	6 (28.6)	5 (23.8)	6 (28.6)	4 (19.0)
Ob	18	13 (72.2)	0	3 (16.7)	2 (11.1)
Os	18	0	2 (11.1)	5 (27.8)	11 (61.1)

*Additional drugs tested were streptomycin, ethambutol, pyrazinamide, kanamycin, amikacin, capreomycin, and ofloxacin.

**Figure 4:**
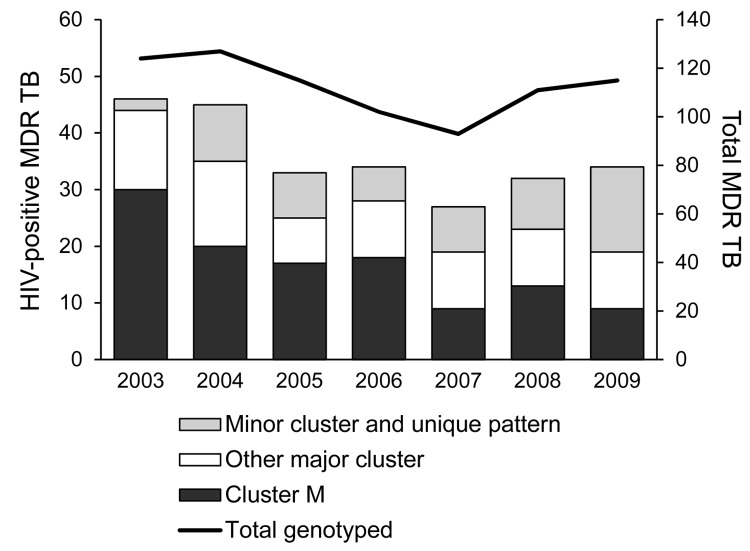
Numbers of HIV-positive patients with multidrug-resistant tuberculosis (MDR TB), classified by genotype cluster, and total number of newly diagnosed MDR TB patients per year with identified genotype, Argentina, 2003–2009.

The predominant cluster, M, was largely confined to the city of Buenos Aires and the surrounding area, with only 5/228 patients having MDR TB diagnosed elsewhere. Twenty patients in this cluster were immigrants from neighboring countries (Bolivia 11, Paraguay 6, Peru 2, Uruguay 1). Most patients had >1 commonly acknowledged risk factors for MDR TB (129 patients had 1, 64 had 2, and 16 had 3 risk factors) (Table 3). The cluster included the 2 previously reported outbreak variants of the M strain (*4*), Mm in 180 patients and Mn in 35 patients (Figure 2), and 9 sporadic variants, observed in 13 patients who had proven epidemiologic links with other patients in cluster M. An isolate resistant to 5 drugs was strongly associated with disease produced by the M strain (Table 5). The numbers of patients affected by this strain decreased significantly within the period when compared with the numbers of patients in the other 6 major clusters (p = 0.002). In particular, the proportion of HIV-infected patients affected by the M strain decreased significantly during the study period, from 65% in 2003 to 24% in 2009 (p = 0.02; Figure 4). No similar trend was observed in the HIV-negative group (p = 0.77).

**Table 5:** Predictors for being in cluster M among 438 patients with multidrug-resistant TB who were in clusters of >15 patients, Argentina, 2003–2009*

Characteristic	No. patients	% Patients in M cluster	Unadjusted OR (95% CI)	Adjusted OR (95% CI)†
Age, y, n = 347				
16–45	288	50.7	0.9 (0.5–1.6)	1.4 (0.5–4.3)
>45	59	52.5	1	1
Country of birth, n = 302				
Argentina	275	51.0	0.7 (0.3–1.6)	0.6 (0.1–2.3)
Other	27	74.1	1	1
HIV status, n = 360				
Positive	194	60.3	**1.6 (1.0–2.4)**	1.4 (0.6–3.3)
Negative	166	49.4	1	1
Previous TB treatment, n = 304				
Yes	160	47.5	1.0 (0.7–1.6)	0.8 (0.4–1.8)
No	144	46.5	1	1
Hospital exposure‡				
Yes	86	72.1	**2.9 (1.7–4.8)**	2.6 (1.0–6.8)
No	352	47.2	1	1
Isolate resistant to				
>5 drugs	207	88.4	**31.5 (18.4–53.9)**	**22.7 (10.1–50.9)**
<5 drugs	231	19.5	1	1

*Boldface indicates significance. TB, tuberculosis; OR, odds ratio. †Overall model fit p < 0.0001. Hosmer & Lemeshow test p = 0.899, area under the receiver operating characteristic curve 0.854. ‡Previous hospitalization(s), concomitant condition, or health care worker.

The second major cluster, Ra, was mainly limited to the overpopulated area of Rosario City and surroundings, another MDR TB hotspot (*7*). Only 8 patients in this cluster were found outside that area. Of 38 patients in the third major cluster, Rb, 26 received a diagnosis of MDR TB in Buenos Aires, including 10 transvestite sex workers; 8 patients had the disease diagnosed in Rosario, and 4 elsewhere. Cluster Pr consisted mainly of inmates in several state prisons for men. An association of gender with clustering was only observed in clusters Rb and Pr, in which men predominated (32/38 and 24/26, respectively).

#### XDR TB Patients and Genotypes

XDR TB was newly diagnosed in 53 patients during 2003–2009. Of these patients, 37 first received a diagnosis of MDR TB during the same period, so these patients were included in the MDR and XDR TB groups. The other 16 XDR TB patients received a diagnosis of MDR TB before this period. The male:female ratio for XDR TB patients was 1.25:1; median age was 37.4 years (SD 11.6, range 21–72 years). Fifty-two patients were born in South America (Argentina 30, Bolivia 4, Peru 6, Paraguay 1, Brazil 1, undetermined 11), and 1 was born in Indonesia.

Characteristics of XDR TB patients related to clustering are described in Table 6. Four major clusters (M, Os, At, and Rb) comprised 31 (58%) XDR TB patients; 6/14 HIV-positive patients with XDR TB were in cluster Os, and 5 in cluster M. Five of 11 immigrants from other South American countries to Argentina harbored the M strain, and the patient from Indonesia harbored a Beijing strain. Of 15 patients with no record of previous TB treatment, 12 were included in major clusters. Annual numbers of XDR TB patients by genotype are shown in Table 7.

**Table 6:** Characteristics of 53 patients with extensively drug-resistant TB, Argentina 2003–2009*

Characteristic	No. patients	% In cluster	% In major cluster
Sex			
M	29	79.3	58.6
F	24	75.0	58.3
Age group, y			
15–29	16	81.3	50.0
30–44	24	75.0	66.7
>45	8	87.5	50.0
Unknown (adult)	5	60.0	60.0
Country of birth			
Argentina	30	76.7	53.3
Other (South America)†	11	81.8	63.6
Unknown (South America)‡	11	81.8	72.7
Indonesia	1	0	0
Place of diagnosis			
Former MDR TB hot spot	31	74.2	71.0
Other	22	81.8	40.9
HIV status			
Positive	14	85.7	78.6
Negative	33	75.8	54.5
Unknown	6	66.7	33.3
Site of disease			
Pulmonary	49	81.6	61.2
Disseminated	3	33.3	33.3
Unknown	1	0	0
Previous TB			
Yes	38	71.1	50.0
No	10	90.0	70.0
Unknown	5	100.0	100.0
AFB smear microscopy			
Positive	41	78.0	56.1
Negative	7	71.4	57.1
Unknown	5	75.0	75.0

*TB, tuberculosis; MDR, multidrug-resistant; AFB, acid-fast bacilli. †From a country in South America other than Argentina ‡From an unknown country in South America.

**Table 7:** Number of patients with extensively drug-resistant tuberculosis of different genotypes, by year, Argentina, 2003–2009

Genotype	No. patients in year							Total no. (%) patients
	2003	2004	2005	2006	2007	2008	2009	
Cluster M	5	4	5	2	0	3	2	21 (39.6)
Cluster Rb	0	0	0	0	0	1	0	1 (1.9)
Cluster At	0	1	0	0	0	0	1	2 (3.8)
Cluster Os	0	0	2	1	2	1	1	7 (13.2)
Minor cluster*	2	1	3	3	1	1	0	11 (20.8)
Unique pattern	2	2	2	1	1	0	3	11 (20.8)
Total	9	8	12	7	4	6	7	53 (100.0)

*Cluster of <15 patients with newly diagnosed multidrug-resistant tuberculosis during the study period

## Discussion

The annual number of newly diagnosed MDR and XDR TB cases decreased slightly, with minor fluctuations, during the study period. HIV infection was associated with almost one third of MDR TB cases; this proportion is 2–4× higher than that attributed by different studies to all forms of TB in the country (*16*,*17*). As previously observed (*18*), annual fluctuations in the numbers of total MDR/XDR TB cases during the study period paralleled closely annual fluctuations in the numbers of HIV-infected patients.

All 7 major clusters in our study were connected with particular geographic areas, institutional settings, or both. Furthermore, most patients in these clusters underwent TB treatment in health centers that had ongoing MDR TB transmission or had a household or a prison contact with persons who had MDR TB. These findings indicate that these major clusters represent true transmission events.

Many cases in this outbreak were caused by the M strain, an apparently autochthonous outbreak genotype. In a countrywide survey performed in 1998, this strain was found to be responsible for 42% of all MDR TB cases but was confined to the metropolitan area of Buenos Aires (National TB Laboratory Network, unpub. data). Since then, the M strain has been the most frequently identified in every MDR TB investigation performed in the country. We found the M strain to be the most prevalent and that its transmission was virtually restricted to the area of the initial outbreak. However, its numbers decreased by more than half within the study period, particularly among HIV-infected patients, which suggests that the epidemic curve of the M strain has entered a declining phase. The other 2 strains associated with previous MDR TB outbreaks, Ra and Rb, were found to persist with lower, fluctuating frequencies.

The expansion of M strain transmission in Argentina was initially fostered by clinical mismanagement. During the early 1990s, patients with advanced AIDS were hospitalized in large, referral treatment centers, where they shared facilities with patients who had MDR TB. At that time, virtually no respiratory protection policy was in force because it was wrongly assumed that MDR TB patients were barely infectious. After genotyping confirmed outbreaks of MDR TB among the patients with AIDS (*4*), hospital infection control interventions were adopted, microbiologic diagnosis and drug-resistance detection were expedited, and second-line TB drugs and highly active antiretroviral therapy became available. As a result of those interventions, hospital transmission was substantially reduced but not completely controlled (*18*). At the time of our study, however, the M strain had long expanded beyond the hospital environment (*6*).

The national *M. tuberculosis* genotype database in Argentina identified very few patients with non–MDR TB harboring H2 genotypes (V. Ritacco, unpub. data). Another study supported this observation (*19*), and the H2 genotype was found to be infrequent in other South American countries (*13*,*20*–*22*). The IS*6110* RFLP pattern of the M strain was absent outside Argentina in the Ibero-American MDR TB genotype database (*23*) and was not present in the *M. tuberculosis* genotype database in the Netherlands (D. van Soolingen, pers. comm.). This pattern was registered in other countries, anecdotally, in 2 MDR TB patients with AIDS: a patient from Argentina who died in San Francisco, California, shortly after his arrival in the United States (*24*); and a patient from Asuncion, Paraguay, who visited the Hospital Muñiz in Buenos Aires each month for antiretroviral therapy (*20*).

Different *M. tuberculosis* genotypes may have affinity with certain geographic areas and ethnic groups (*25*), which could explain why the M strain persists in the Buenos Aires area. In addition, most persons at risk for MDR TB are underprivileged and cannot afford to travel far distances. This mobility limitation might also help to explain why this strain has remained virtually confined to the original hotspot area.

A small number of patients affected by the M strain were immigrants from neighboring countries who had settled in Buenos Aires. Cross-border and domestic migration toward large metropolitan areas is a long-observed demographic and public health concern in Argentina. More than 80% of the patients in this study were assisted in metropolises designated as MDR TB hotspots. Even though immigrants with TB have access to higher quality health care in these areas than in other parts of the country, they are also at higher risk of becoming newly infected with an outbreak MDR TB strain.

The M strain was overrepresented among patients with XDR TB; isolate resistance to >5 anti-TB drugs was found to be a strong predictor of disease caused by the M strain (*4*,*26*). The accumulation of drug resistance–conferring mutations would be expected to have reduced the epidemiologic fitness of this strain, but it has prevailed for 15 years. The epidemiologic fitness of a strain can be influenced by a range of factors, e.g., the genetic backgrounds of host and pathogen, host–pathogen interactions, and the environment (*27*–*29*). Compensatory evolution restoring in vivo fitness, as well as social and behavioral factors, might have played a role in the epidemiologic persistence of the M strain (*30*). These factors might also have preferentially fostered the spread of drug-resistant strains of the H2 genotype in our setting. Further studies are needed to evaluate the most critical risk factors.

Drug-resistance profiles were not uniform within the M strain clusters. The variations in susceptibility to individual drugs reflect the existence of various ongoing chains of transmission, some of which might have started before, or simultaneously with, the first documented outbreak. One limitation of our study is the failure to identify individual chains. Factors that precluded the reliable characterization of subclusters were the long time elapsed since the outbreak onset, the insufficient epidemiologic documentation in many cases, and the unavailability of additional molecular markers.

Our study has another major limitation. Incomplete demographic and clinical data on patients were retrieved, and several observations had missing values. If missing values were systematically associated with a given force or factor, results presented here would be biased. We are not aware of any association of missing values with the dependent variables under study and assume that those data were missing at random. Missing values may have affected the analyses by reducing the number of observations, which may have reduced the power of the model to detect significant associations but without necessarily biasing the associations reported. However, the possibility that bias might have resulted from missing data cannot be ruled out. Therefore, statistical significances of our analyses should be interpreted cautiously.

In the MDR TB hotspots in Argentina, the distinction between primary and acquired MDR TB on the basis of a history of previous TB treatment was not decisive because patients could have been exposed to hospital-associated MDR TB infection while being treated for community-acquired TB. This fact could explain why clustering was not more frequent among patients without previous TB treatment in our study.

The national TB network includes all the laboratories performing bacteriological TB diagnosis in the country; therefore, the patients in this study represent all newly diagnosed MDR TB cases in Argentina. The structure, geographic coverage, and personnel of the TB laboratory network are adequate to provide DST for all patients at risk for MDR TB in Argentina. However, a few MDR TB patients might remain undiagnosed because of operational factors, e.g., inefficient detection of risk factors, insufficient or delayed requests for DST, and disorganized information systems.

The geographically restricted distribution of successful MDR TB genotypes that we found has public health implications. As a result of this study, specific interventions are being reinforced, particularly in the MDR TB hotspots: implementing universal culture and strategies to expedite drug resistance detection; decentralizing specialized health care; streamlining information-sharing systems between HIV and TB programs; and strengthening administrative infection control measures in prisons and large hospitals with high TB infection load. A national advisory group on MDR TB clinical management has also been recently created. Control interventions have already started to reduce MDR TB spread in the hospital that was the epicenter of the main outbreak (*17*). Still, centrally coordinated actions are needed in Argentina to curb long-term transmission of MDR TB.
